# A Review of MEMS Capacitive Microphones

**DOI:** 10.3390/mi11050484

**Published:** 2020-05-08

**Authors:** Siti Aisyah Zawawi, Azrul Azlan Hamzah, Burhanuddin Yeop Majlis, Faisal Mohd-Yasin

**Affiliations:** 1 Institute of Microengineering and Nanoelectronics, Universiti Kebangsaan Malaysia, Bangi, Selangor 43600, Malaysia; aisyahzawawi@gmail.com (S.A.Z.); azlanhamzah@ukm.edu.my (A.A.H.); burhan@ukm.edu.my (B.Y.M.); 2 UiTM Foundation Centre, Universiti Teknologi Mara, Dengkil Campus, Dengkil, Selangor 43800, Malaysia; 3 Queensland Micro- and Nanotechnology Centre, Griffith University, Brisbane QLD 4111, Australia

**Keywords:** micro-electro-mechanical system (MEMS), capacitive microphone, condenser microphone, acoustic sensor, evolution

## Abstract

This review collates around 100 papers that developed micro-electro-mechanical system (MEMS) capacitive microphones. As far as we know, this is the first comprehensive archive from academia on this versatile device from 1989 to 2019. These works are tabulated in term of intended application, fabrication method, material, dimension, and performances. This is followed by discussions on diaphragm, backplate and chamber, and performance parameters. This review is beneficial for those who are interested with the evolutions of this acoustic sensor.

## 1. Introduction

Ever since the introduction of modern microphone back in the late 19th century, tremendous progress had been made due to its broad and evolving list of applications in consumer [1,2], medical [3,4], and automotive applications [5,6]. Johann Philipp Reis and Alexander Graham Bell are acknowledged as the inventors of the first microphones [7]. These early prototypes produced electrical signals with low amplitude and limited frequency ranges. As a result, the sound quality was so low that the devices were barely capable of reproducing intelligible speech. The development of the functioning microphones was credited to Thomas Edison, Emile Berliner, and David Edward Hughes [8]. Their carbon microphones later dominated the markets. Edison and Berliner separately announced their inventions (called transmitters back then) and fought over the patent right in the United States. In the United Kingdom, Hughes demonstrated similar device to the Royal Society in London and coined the term microphone, although he did not apply for a patent. The telecommunications industry quickly realized the potential of microphones in their systems and pushed for technological innovations. The variants of the carbon microphone were commonly used in telephone between 1920s and 1980s. Riding on the rapid growth of telecommunication and music industries, other forms of transduction mechanisms continue to be developed and employed in the telecommunication systems. A capacitive microphone was introduced in 1916 and currently dominates the markets. The newest kinds are the optical-based and spintronic microphones.

There are a variety of transduction mechanisms being used in microphones to convert acoustic waves to electrical signal, such as electromagnetic (electrodynamic), piezoresistive, piezoelectric, optical, spintronic, and capacitive. For the first mechanism, an electromagnetic microphone consists of a coil that moves through a fixed magnetic field to produce the alternate current, i.e., electrical output. The coil is attached to a thin diaphragm that vibrates according to the acoustical input. The carbon- and ribbon microphones are the variants of this type [9,10,11]. An electromagnetic microphone has a sensitivity issue due to the slow vibrating velocity of heavy diaphragm and coil [12]. The second mechanism is called piezoresistive microphone. It operates as follows. On top of an acoustic diaphragm, there are four resistors that are connected in a Wheatstone bridge. When the pressure is induced by the sound waves, the diaphragm deflects. Accordingly, the stress-dependent values of the four resistors changes, as well. The Wheatstone bridge produces an output voltage based on the difference between the values of these resistors. Piezoresistive microphone has the disadvantages of relatively low dynamic range and sensitivity [13] but is nevertheless being used in many applications. The third mechanism is called piezoelectric microphone. It uses the principle of piezoelectricity to convert mechanical vibrations from sound waves to electrical signals [14]. For this purpose, aluminum nitride and zinc oxide are the common piezoelectric materials that researchers used to fabricate the thin diaphragm. Due to the rigidity of this material, this type of microphone is originally used to amplify contact-based vibration from musical instruments. Due to its advanced performances today, it has more diverse applications. As for the fourth mechanism, an optical or fiber-optic microphone uses light source to illuminate the thin diaphragm. A photo detector is used to detect the light’s intensity and wavelength. When the diaphragm is vibrated by the acoustic waves, the difference between the original and the reflected sources is recorded and further converted to electrical signal. Optical microphone’s main strength is that it is not susceptible to electrical noise and electromagnetic interference. The disadvantage is the complexity of the detection system, which translate to higher cost. It has niche markets in medical applications and high-fidelity recordings. Spintronic microphone is the fifth mechanism, which is based on magneto-resistance transduction. It is proposed to solve the low sensitivity issue that haunts piezoresistive microphone. The spin strain gauge sensor (SGS) replaces resistors on top of the acoustic diaphragm. This spin SGS is highly sensitive as it is based on magnetic tunnel junction effect. This approach was recently proposed by the Japanese researchers [15,16,17,18]. The sixth mechanism is called condenser microphone or more commonly known as capacitive microphone. It functions via changes in capacitance between movable and fixed plates. The thin diaphragm represents the movable plate. Incoming sound waves vibrate it, thus proportionally varying the value of the capacitance. A voltage source is needed to bias the plates at a fix voltage. The electret microphone is a specific type of capacitive microphone that keeps a permanent bias between its plates by means of an electret material. Due to its good performance, low cost, and ease of manufacture, the electret microphone became the most commercially manufactured microphone type with over one billion units produced annually at the height of its production [19]. 

A micro-electro-mechanical system (MEMS) microphone, which is the micro-scale version of the microphone, gained its footing in the commercial mobile market in the early 2000s, mostly due to the cost and footprint. Since its inception into mobile devices, the market began to skyrocket. The main driver for its rapid growth is because major phone manufacturers are putting as many as eight MEMS microphones into a single mobile device. In addition to acoustical-to- electrical signal conversion, this device is very versatile and could be used to replace other expensive sensors. For example, an array of MEMS microphones can be programmed to map an acoustical image [20,21] and moving vehicle [22]. Another future application is as proximity sensor, by setting the MEMS microphone to operate in the ultrasonic range. It can sense finger movements hovering a few millimeters above the mobile phone’s touchscreen to avoid physical contact. As a result, the phone’s screen will not get dirty and greasy [23]. 

In addition to mobile phones, electronics manufacturers are integrating MEMS microphones and other sensors into various handheld devices, smart watches, tablets, medical devices, wearable electronics, and Internet of Things (IoT) devices. Jérémie Bouchaud and Marwan Boustany of IHS Markit analyzed consumer and mobile MEMS markets from 2010 to 2018 [24]. They highlighted three important trends. First, the growth of MEMS market has expanded from slightly below $2 billion USD in 2010 to over 5 billion USD in 2017. Second, the top three products that employ MEMS sensors are mobile handsets, media tablets and wearable electronics. Third, all these devices heavily employ MEMS microphones. This market push solidifies the importance of MEMS microphone for years to come. 

One of the earlier adopters of MEMS microphone is Apple, Inc. This company has been rigorously incorporating MEMS capacitive microphones into their iPhone products since the development of the iPhone 4 [25]. Knowles Technology and Infineon Technologies won the design for the three MEMS microphones incorporated in the iPhone 4, two for primary audio sensing and one for background pickup for noise cancelation. Knowles’ S4.10 and S2.14 microphones have a circular top movable diaphragm diameter of approximately 0.5 mm. The size is sufficient to capture sound wavelength, which is approximately 34 mm at 10 kHz. Both have die area of less than 1.6 mm^2^, with either two or four leads for interconnections. Knowles microphones utilize damping holes that co-functions as etch release vents during the fabrication process. Besides Knowles, Infineon Technologies provided the E2002 MEMS microphone for the iPhone 4. It has circular diaphragm with the diameter of 1 mm. Since the iPhone 4, capacitive microphones from Knowles and Infineon, along with microphones from other key manufacturers, such as Analog Devices, have won design contracts for various subsequent Apple products, including later generation iPhones and the iPod Nano. For example, Analog Devices designed the ADMP 403 MEMS microphone for the 5th generation iPod Nano. They proposed a circular diaphragm with a diameter of 0.59 mm. 

**Figure 1:**
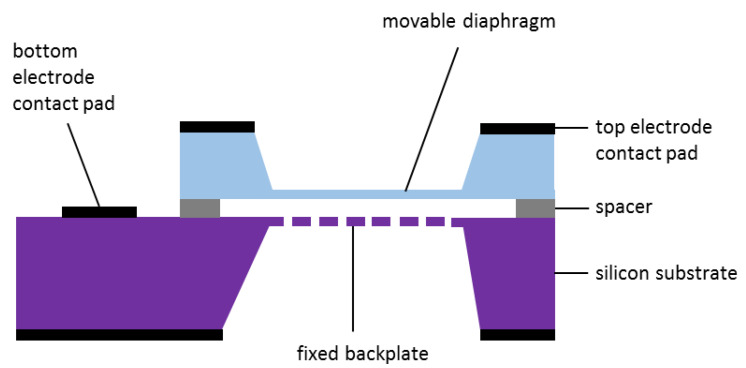
Basic structure of a micro-electro-mechanical system (MEMS) capacitive microphone.

The basic structure of MEMS capacitive microphone is shown in Figure 1. It consists of two parallel plates, i.e., movable top diaphragm and fixed backplate. Both are separated by an air gap, and an insulator was used as the spacer. The top and bottom plates are connected to separate electrodes that measure the output signal. The diaphragm vibrates when acoustic pressure is applied onto it, hence producing variation in the air gap. The resulting parallel plate capacitance is given by Equation (1):

(1) C=εoA/g,

where C, εo, *A*, and *g* is capacitance, permittivity of the dielectric material, area of the plate, and air gap, respectively. The value of the measured capacitance is proportional and inversely proportional to the size of the diaphragm and air gap, respectively.

The perforated backplate is shown as a dashed line in Figure 1, of which the dashes represent acoustic holes. They enable air to stream in and out of the air gap when the diaphragm vibrates. Without these holes, the squeezed air between the diaphragm and backplate becomes a mechanical dampener. It reduces the ability of the diaphragm to vibrate, especially at higher frequency. In other words, the sensitivity of the microphone will be greatly reduced. The damping resistance can be expressed using Skvor’s formula [26,27] as:

(2) $$R_{ag}=\frac{12\mu }{n\pi g^{3}}B\left(Ar\right),$$

where μ=18.6×10*−*$6\;\mathrm{Ns}/m^{2}$ (viscosity of air), L= hole length (backplate thickness), n= number of holes, g= air gap thickness, and B(Ar) is a coefficient of the effective backplate area. The latter is given as:

(3) $$B\left(Ar\right)=\frac{1}{4}ln\left(\frac{1}{Ar}\right)-\frac{3}{8}+\frac{1}{2}\left(Ar\right)-\frac{1}{8}{\left(Ar\right)}^{2},$$

where (Ar) is the ratio of hole area to non-hole area. Equation (3) reveals that by increasing air gap and number of perforated holes, the mechanical damping is reduced. The open circuit sensitivity of MEMS microphone is given by Equation (4) [28]:

(4) $$S=S_{e}\times S_{m}=\frac{V_{b}}{g}\times \frac{\Delta g}{\Delta P},$$

where $S_{e}\;$ and $S_{m}$ are electrical and mechanical sensitivity, respectively, while $V_{b}$, g, Δg, and ΔP are bias voltage, air gap, change in air gap, and change in pressure, respectively. Three important relationships could be deduced from Equation (4). First, the electrical sensitivity depends on the value of the bias voltage and the thickness of the air gap. Second, the mechanical sensitivity depends on the changes in air gap and pressure. Third, the open circuit sensitivity is the product of $S_{e}$ and $S_{m}$. It is important for the designer to tackle both in order to get higher sensitivity. 

Finally, we should also observe the relationship between the dimension and stress of the diaphragm to the mechanical sensitivity ($S_{m})$ of the microphone [29], as shown in Equation (5):

(5) $$S_{m}=\frac{R^{2}}{8\sigma_{d}t_{d}\;},$$

where *R* is the radius of the diaphragm (for circular shape), and $\sigma_{d}$ and $t_{d}\;$ are the stress and the thickness of the diaphragm, respectively. It is clear from this equation that the designers must be careful with the choice of material and the size of the diaphragm to increase the performance of their devices.

The main purpose of this article is to provide a state-of-the-art review on advances in the MEMS capacitive microphone based on the academic papers that were published in open literature. The first review paper on MEMS microphone was written by Scheeper et al. in 1994 [29]. That highly cited article masterfully covered the theoretical parts, such as the sensor’s mechanical and electrical sensitivity, frequency responses, and electrical noise. The equations are still valid and being used today. Section 4.2 of that review discussed the design and fabrication of electret and capacitive microphones from 1984 to 1993. There have been a lot of new developments since then, but there has been no subsequent review until recently. In 2018, Ishfaque et al. [30] published their review paper on the silicon microphone. However, these authors only focused on the advances in directional microphones that are inspired by the parasitic fly called *Ormia Ochrasia*. It was pioneered by Miles et al. in 1995 [31] but has not been widely commercialized. We will not discuss that specific type of MEMS microphone in this review. In 2019, Shah et al. [32] presented a wide review of MEMS microphones, covering different types of transduction mechanisms and using data from academic papers and commercial products. In this paper, we offer a focused review on the MEMS capacitive microphone. It contains detailed summaries of all academic papers from 1989 to 2019. These are tabulated and organized in the form that is easily referred to by readers and future MEMS capacitive microphone designers. It should be noted that the design of the electrical amplifier is not covered in this review, as it is treated as separate module. Earlier works from Kuhnel et al. [33] and Graf et al. [34] attempted to integrate the microphone with an electrical amplifier made of a field effect transistor. They found that the noise floor level is much higher than microphone without the amplifier [35]. 

The rest of the paper is as follows. Section 2 compiles all the published articles that showcase significant developments in capacitive microphone. This is the “crown jewel” of this review. Section 3 and Section 4 discuss the details of diaphragm, backplate, and back chamber, respectively, while Section 5 discusses the parameters that limit microphones performances. Finally, Section 6 discusses the outlook on the development of MEMS capacitive microphones, and Section 7 conclude this paper. 

## 2. Summary of Academic Papers on MEMS Capacitive Microphones

Table 1 lists the published papers on MEMS capacitive microphone in the past 30 years. Most of them have been published in leading journals and conferences. Each row in the table represents different papers in chronological order. The columns consists of five segments with the following parameters: Column 1 (author and year of publication); Column 2 (niche application and key fabrication method); Column 3 (diaphragm properties: material, geometry, size, thickness, air gap); Column 4 (backplate properties: electrode material, backplate material, and damping reduction mechanism); and Column 5 (performance of microphone: bias voltage, stray capacitance, input sound pressure level (SPL), open circuit sensitivity, and resonance frequency). 

Based on the information from Table 1, the widely used fabrication method are bulk micromachining. This process produces structures inside a substrate, which can be patterned using anisotropic etching, isotropic etching, or reactive ion etching (RIE). The second method is surface micromachining, which produces structures by depositing layers on top of the substrate. This is more efficient method in comparison to bulk micromachining, but also more complex. The third option is using a complementary metal-oxide-semiconductor (CMOS) process, which was originally used for integrated circuits. In order to be used to make microphone, CMOS’s metal-dielectric layers are employed. This is perhaps the cheapest option among all three methods. able 1 showcases different materials, designs, and dimension that researchers employed to make diaphragms. Among the deciding factors are the ease of fabrication, management of residual stress, and performances. Furthermore, these authors differ on the materials of backplate and dimensions of the back chamber, as they play an important role as a damping reduction mechanism. Many researchers introduced acoustic holes on the backplate and varied the size of the back chamber to increase the sensitivity of their prototypes.

Table 1 serves as the main source of information for academic research in MEMS capacitive microphones. Readers could use the information that is collated to analyze the evolution of this device in the past 30 years. New researchers in this topic could predict the performances of their planned prototypes based on the closest design, material, bias voltage, and dimensions. The remaining sections of this review explain the design components and performance parameters that are listed in Table 1. 

## 3. Acoustic Diaphragm

The diaphragm is the most important part of a MEMS capacitive microphone as it senses induced pressure from the sound waves. In Section 3.1, we highlight the groups that pioneered the use of these materials. They have different properties, such as Young’s Modulus, Poisson ratio, coefficient of thermal expansion, thermal conductivity, and density. Section 3.2 covers the design and fabrication of diaphragm’s geometry and surface topologies. Early MEMS microphones employed a square diaphragm with a flat surface, as they were easy to fabricate. We then narrate the efforts by later groups for circular diaphragms with corrugated topology. Finally, Section 3.3 covers several groups that attempted to control the residual stress of their diaphragms. This effort is importance for reliability and sensitivity of the device.

### 3.1. Materials

Hohm et al. was the first group that fabricated a MEMS capacitive microphone. Their first choice for diaphragm was actually mylar film [129]. However, they noted that it wrinkled under compressive stress. Then, they employed Si_3_N_4_ as a diaphragm material with better tensile stress [36]. Afterwards, other research groups followed in their footsteps by developing SiN diaphragms with various geometries and topologies. The most notable group is Scheeper et al. [39,43,69,130]. This is the one that authored the first review paper on silicon microphone in 1994 [29]. 

Some researchers employed Si or poly-Si as diaphragm materials because of ease of fabrication. Bergqvist et al. is the pioneer in utilizing the Si diaphragm. This group produced their first prototype in 1990 [37], with follow up works after few years [41,42]. Later, a group of Japanese researchers perfected their design of single crystal Si microphone [70,82,83]. Instead of pure Si, many researchers turned to poly-Si for cost and easier patterning. Zou et al. is one of the first that used poly-Si to make a corrugated diaphragm [48]. This complex geometry is said to reduce the built-in stress and mechanical stiffness. Two groups separately published their microphones using a poly-Si diaphragm in 2000. Buhrdorf et al. announced their ultrasonic transducer, which was an effect on a microphone that is capable of operating up to 500 kHz [55]. Torkkeli et al. [56] had a fancier perforated diaphragm, although both groups utilized square geometry. Brauer et al. came up with circular poly-Si diaphragm in 2001 [57]. 

There are several groups that employed metals as the diaphragm. These have low mechanical sensitivity but are easier to pattern. Lee et al. [93] employed a gold (Au) circular diaphragm for their electroacoustic transducers. This group attempted to demonstrate the feasibility of using standard CMOS process to make a microphone, where Au is one of the interconnect materials. Ganji et al. experimented with a square-shaped aluminum (Al) diaphragm. They choose Al to make the perforated diaphragm [91,131], as it has a low Young’s Modulus (70 GPa). The same group also patterned a slotted Al diaphragm [92,95]. Slot is defined as a long lines of emptied space, which achieved the same effects as perforated holes. In a follow up work from another group in Taiwan, Huang et al. developed a circular corrugated Al diaphragm fabricated from the CMOS 0.35 µm process [100]. The latest work on an Al-based diaphragm is by Sedaghat et al. [121], in which they attached a “frog arm” spring to it. 

Finally, two groups had attempted to use polymer as diaphragm. Sim et al. [65] employed parylene-C and study the effect of stress on flat and corrugated diaphragms. In addition, Pedersen et al. [51] proposed a polyimide diaphragm made directly on the substrate by micromachining process. The main advantage of this material is the low temperature (<300 °C) needed in fabrication process. The main disadvantage is that polyimide is not a good conductor. Nevertheless, the group was able to develop their prototype, achieving open circuit sensitivity of 10 mV/Pa. 

### 3.2. Geometry and Surface Topology

**Figure 2:**
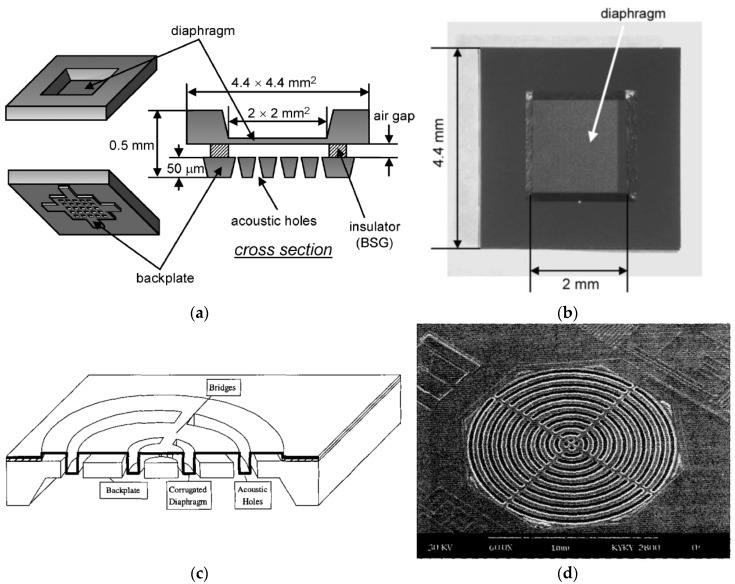
(**a**,**b**) The schematic and top view of fabricated flat diaphragm, respectively, from Goto et al. [82]. (**c**,**d**) show the schematic and SEM image of corrugated diaphragm from Chen et al. [66].

All MEMS microphone pioneers in 1980s and 1990s started with a flat diaphragm, despite using different materials, because of its simple fabrication processes. Later, researchers realized that their diaphragms had to be patterned to control the residual stress. The next evolution after the flat diaphragm is the corrugated diaphragm, as it can reduce the compressive stress, hence increasing the open circuit sensitivity. Scheeper et al. is the pioneer by making corrugated diaphragm from SiN in 1994 [130]. After that, other groups started to follow through. Zou et al. proposed their corrugated diaphragm using poly-Si in 1997 [48], which was followed by Kressmann et al. [64], Chen et al. [68], and Wang et al. [71,74,75]. Wang group must be credited for their thorough investigation of the sensitivity studies of single corrugated poly-Si diaphragm. Two key observations are as follows. First, circular corrugated diaphragm gives higher open circuit sensitivity compared to square corrugated diaphragm. Second, larger corrugation depth led to higher sensitivity. The designs for flat and corrugated diaphragms are shown in Figure 2.

### 3.3. Stress of the Diaphragm

In essence, the stress of the diaphragm depends on the choice of materials. Developers favor tensile over compressive stress for higher sensitivity. Miao et al. [67] suggested that it could be tuned by monitoring the process parameters during the fabrications of diaphragm, such as higher annealing temperature and lower base pressure. Some groups employed implantation method on the material, for example N_2_ ion [36], boron ion [42,56,73], and phosphorous ion [27,88]. These ion implantation changes the stress gradient of the diaphragm due to the mismatch between the coefficients of thermal expansions (CTE) of each material. 

The second method to reduce stress is by patterning the diaphragm, as detailed in Section 3.2. The third method is by utilizing spring to suspend the acoustic diaphragm from the body. The conventional designers use edged-clamped diaphragms. The ability of the diaphragm to deflect is determined from its effective spring constant (*k)*. The value of *k* can be determined from the deflection of the diaphragm using Equation (6) [56]:

(6) $$k=8\pi \sigma_{d}t_{d},$$

where $\sigma_{d}$ is tensile stress in Pascal (Pa), and $t_{d}$ is the diaphragm thickness in meter (m). Several groups attached spring(s) or hinge between their diaphragms and the backplates. It enables the diaphragm to have greater flexibility to release and absorb stress. This method were proposed by Weigold et al. [79], Kim et al. [81], and Mohammad et al. [97], among others. 

## 4. Backplate and Back Chamber

The second most important parts of MEMS microphone are the backplate and back chamber. They determine the dimension of the prototype and the distance of air gap with the acoustic diaphragm. In addition, there must be outlet valve to control the air damping inside the chamber. It should be mentioned that the latest design from two groups, i.e., Lo et al. [113] and Mao et al. [118], introduced MEMS capacitive microphone without a backplate. In this structure, the perforated diaphragm and its substrate are the top and bottom plates, respectively.

Nevertheless, most groups follow the conventional structure, which necessitates this section. In Section 4.1, we describe the type of materials that are being used to make backplate and the justification for choosing them. Then, the next two sub-sections discuss the mechanisms to reduce the effect of air damping. Section 4.2 highlights works that etched acoustic holes onto their backplate, while Section 4.3 discusses two works that increased the volume of their back chambers. After that, Section 4.4 discusses air gap and efforts by several groups to study its effect to the sensitivity of their devices. We also highlight the initiative by one group that created “stopper” to avoid the diaphragm and backplate from touching each other. Finally, Section 4.5 briefly covers the materials that were chosen as the electrodes.

### 4.1. Backplate Materials

The capacitance of the microphone (C) is determined by the ratio of charge (Q) and bias voltage (V) being applied on both plates. Clearly, the types of material play an important role in order to produce sufficient Q. Metal is the best conductor, but semiconductor materials are an abundance in MEMS fabrication processes. The positively charged diaphragm (p-type semiconductor) and negatively charged backplate (n-type semiconductor) act as positive and negative terminals, respectively. Therefore, as can be seen from Table 1, most researchers employed Si and poly-Si as backplate material [39,43,47,67,88,95]. Few groups did opt for metals instead. For example, Buhrdorf et al. [55] employed perforated nickel (Ni), and Kabir et al. [54] proposed perforated gold (Au) for their backplates.

### 4.2. Acoustic Holes

**Figure 3:**
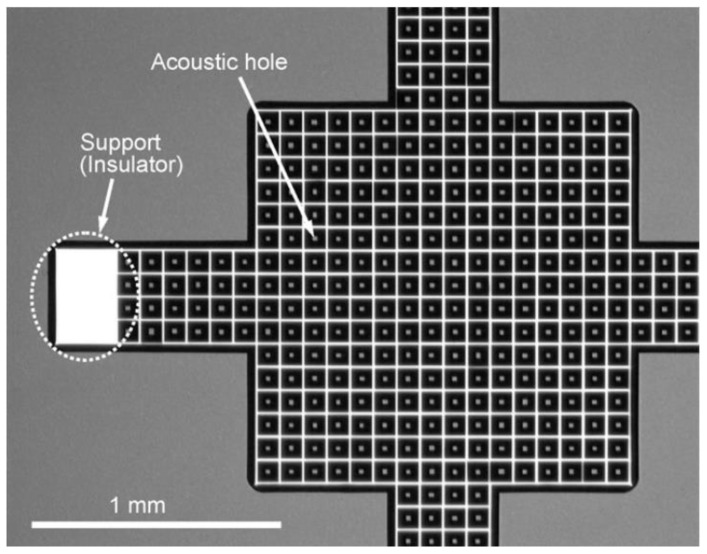
Iguchi et al. [83] systematically etched 10 µm × 10 µm^2^ acoustic holes on 2 × 2 mm^2^ Si (100) backplate.

Table 1 states that most research groups had perforated backplates to reduce the air-streaming resistance due to vibration being induced by the movable diaphragm. This is accomplished by etching acoustic holes on that plate. It should be mentioned that the same effect could be accomplished by etching the holes in the diaphragm. For example, Ganji et al. [91,131] did that on Al diaphragm. However, majority of groups prefer to etch backplate as it is thicker, and therefore easier to pattern. We would like to highlight one good design example from Iguchi et al. [83]. The optical microscope photograph of their backplate is shown in Figure 3. The dimension of the square backplate is 2 × 2 mm^2^, and it is 50 µm thick. It can be seen from the figure that 10 µm × 10 µm^2^ acoustic holes were systematically etched on the Si backplate. A square, instead of circular, hole is patterned due to the ease of anisotropic etching. 

Recent groups attempted to produce circular acoustic holes. For example, Lee et al. [116] employed a total of 1962 circular holes on the backplate that is 0.65 mm in diameter. Each hole has a radius of 4 µm. 

### 4.3. Volume of Back Chamber

The volume of the back chamber is determined by the area of the backplate and the distance of the air gap. This space is unsealed and is filled with air. When the acoustic diaphragm vibrates due to induced pressure from the sound waves, the air inside the chamber acts as resistance and dampen the vibration, especially at high frequency. Equation (3) in Section 1 models it as mechanical resistance. One of the key parameters that determine the size of this resistance is the volume of the back chamber. As the size of back chamber increase, the effect of the air damping is reduced, as air has bigger space to travel. Few groups experimentally verified this relation. Torkkeli et al. [56] reported that, as the volume of the back chamber increased from 0.8 to 100 mm^3^, the sensitivity of microphone went up to 4 mV/Pa. The same effect has recently been observed by Grixti et al. [111].

### 4.4. Air Gap

Most of the works in Table 1 employed an insulator, such as SiO_2_, as an air gap. It was deposited as a sacrificial layer to form a cavity between the diaphragm and the backplate. One group did something different. Shin et al. [113] etched a Si substrate to create the air gap for their prototype. As stated by Equation (5) in Section 1, air gap between the top and bottom plates determines the open circuit sensitivity of the capacitive microphone. Table 1 shows that there is no magic number; all groups employed varying distances that suited the intended sensitivity of their prototypes. One group in particular, i.e., Tajima et al. [70], experimentally verified that air gap is inversely proportional to the sensitivity. They also found that at least 10 µm of air gap is required to achieve stable operating microphone, achieving 10 mV/Pa of sensitivity. Table 1 lists the distance of air gap for other works, as well. 

**Figure 4:**
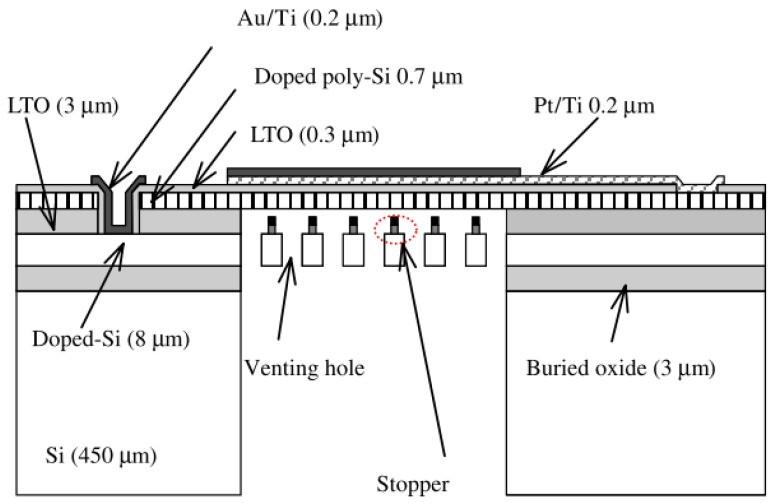
Ko et al. [77] introduced a “stopper” that is attached to the backplate. In the event that the diaphragm vibrates at large amplitude, the stopper prevents it from touching the backplate.

If the amplitude of the induced pressure is very large, the diaphragm could touch the backplate, hence creating a short circuit between both electrodes. In order to mitigate this, Ko et al. [77] introduced stoppers on the backplate. The stopper design is shown in Figure 4. 

### 4.5. Electrodes

The positive and negative electrodes play important role in connecting diaphragm and backplate to the output signal. Since both plates are normally made of semiconductor materials, bond pads are deposited to connect them to the electrodes. Table 1 list varieties of metals that had been chosen to function as the connector (or sometimes referred to as wire or interconnect). Al seems to be the favorite choices in majority of works, as it is in abundance and can be easily sputtered on top of the MEMS structure. Several groups opted for more expensive Au, or its variant of Ti/Au and Cr/Au, because it has higher conductivity.

## 5. Parameters that Determine Performances of MEMS Capacitive Microphones

The last columns in Table 1 list the key parameters that are used to measure the performances of the capacitive microphones, namely bias voltage, stray capacitance, input SPL, open circuit sensitivity, and resonant frequency. We describe them in separate sub-section here, as well as the impact that they impose on the microphone. Where possible, we give average values based on Table 1 and highlight the works of some groups that recorded extraordinary results.

### 5.1. Bias Voltage

The bias voltage is allegedly the easiest parameter to be modified, as Equation (5) dictates, that it is proportional to the electrical sensitivity. Unfortunately, this is not true, as increasing this parameter will eventually collapse the diaphragm to the backplate. The maximum voltage when this happens is called pull-in voltage, which is given by Equation (7):

(7) $$Vp=\sqrt{\frac{8}{27}\frac{kg_{o}{}^{3}}{\varepsilon_{a}A_{e}}},$$

where *k* is the effective spring constant of the diaphragm as given in Equation (6), $g_{o}$ is the air gap at bias voltage of zero, $\varepsilon_{a}$ is the permittivity of air, and $A_{e}$ is the effective area of the diaphragm minus the acoustic holes. In order to avoid the collapse, the rule of thumb is to set the bias voltage to be one third of pull-in voltage. Two groups, i.e., Ganji et al. [87] and Kuntzman et al. [108], pushed the limit of their prototypes by using a bias voltage ≥100 V. The low values of open circuit sensitivity of 0.2 and 0.167 mV/Pa reveal their need for such high voltage. Otherwise, as can be seen in Table 1, most groups opted for more reasonable values below 20 V.

### 5.2. Stray Capacitance

Stray capacitance should not have been confused with the output capacitance (C) that is mentioned in Equation (1). The latter is the output that is measured from the diaphragm and backplate’s electrodes. The former is a parasitic capacitance that present between both plates to other conductive materials, such as bond pad and anchor. In the circuit model, stray capacitance is added to the measured output capacitance from the backplate, hence decreasing the accuracy of the output. Therefore, many groups attempted to minimize it. The guiding principle is to minimize the potential difference between these conductive materials and the diaphragm/backplate. As can be seen in Table 1, several groups managed to reduce the values as low as 0.2 pF [104], 0.7 pF [100], 2 pF [83], 2.12 pF [91] and 2.4 pF [106].

### 5.3. Input SPL

Input sound pressure level (input SPL) is the ratio between the surrounding audible sound (which is measured by sensing its pressure) and the lowest pressure that can be detected by human ears. It is given by the following equation:

(8) $$Input\;SPL=20\log\frac{p_{1}}{p_{2}},$$

where $p_{1}$ is a sound pressure, and $p_{2}$ is a reference sound pressure (20 µPa). The normal sound pressure for human speech is in the range of 60 dB SPL to 70 dB SPL, while the auditory threshold for human ears is 20 µPa (or 0 dB SPL). Researchers use input SPL to characterize the maximum pressure ($p_{1})$ that their prototypes could detect. The input SPL column in Table 1 refers to this value. The highest recorded sound pressure was by Martin et al. [27] with the value of 164 dB SPL. The average values were around 120 dB SPL [51,52,57] and 122 dB SPL [82], while the lowest was 24 dB [60]. In some works, researchers use the standard reference input signal of 1 KHz sine wave at 94 dB input SPL (or pressure of 1 Pa) as $p_{1}$ to find the sensitivity of their microphone. For this case, 94 dB is recorded as input SPL. 

### 5.4. Open Circuit Sensitivity

As stated by Equations (2) and (5), the open circuit sensitivity can be increased by modifying the following parameters: bias voltage, air gap, area of diaphragm, diaphragm thickness, and diaphragm stress. Looking at Table 1, the sensitivity of 10 mV/Pa seems to be a good benchmark. Section 3 and Section 4 already describe the efforts by many groups to increase the value this parameter in term of the material and topology of the diaphragm, as well as perforated backplate, volume of the back chamber, and reducing air gap. In order to avoid duplication, those strategies are not repeated here. 

### 5.5. Resonant Frequency

The resonant frequency (*f_o_*) limits the upper bandwidth of the microphone. It is given as [132]:

(9) $$f_{o}=\frac{1}{2\pi }\left[\frac{\frac{8\pi^{2}Et_{d}{}^{3}}{9\left(1-\mu^{2}\right)a^{2}}+\frac{\kappa^{-1}a^{2}}{18g}}{\frac{\rho t_{d}a^{2}}{10}}\right],$$

where *E, µ*, and *ρ* are Young’s Modulus, Poisson’s ratio, and density of material, respectively, *κ* is compressibility of air, g is air gap between the plates, and $t_{d}$ and *a* are thickness and side length of square diaphragm, respectively. Equation (8) shows that *f_o_* is affected by properties of the material and the dimension of the diaphragm. Although this equation is designed for square diaphragm, it can be used for circular diaphragm by assuming equal areas, given in Equation (9) [64]:

(10) $$a^{2}=\pi R^{2},$$

where *a* is a side length of square diaphragm, and *R* is a radius of circular diaphragm.

Table 1 shows the values of *f_o_* from all the works. Most researchers designed their capacitive microphone on human hearing range. Therefore, it is not surprising that most works had *f_o_* ≤ 20 kHz. For example, Martin et al. applied their microphone for aeroacoustic measurement with frequency range from 300 Hz to 20 kHz [27]. Several groups designed their prototypes as hearing aid devices with different *f_o_*, i.e., 4 kHz [80], 10 kHz [106] and 14 kHz [43].

However, several group custom-made their prototypes for different *f_o_* to cater for specific applications. For example, Hohm et al. developed their microphone to have a very low *f_o_* of 2 kHz for airborne sound detection [36]. On the other extreme, Hansen et al. proposed a capacitive micromachined ultrasonic transducers (CMUTs) at frequency range of 100 Hz to 100 kHz for the generation and reception of ultrasound in air and water [72].

### 5.6. Noise Floor

Another parameter that affect the performance is the noise floor. Not many groups reported this parameter in their articles, hence, it is not included in Table 1. The squeezed-film effect due to air damping is the dominant noise mechanism. For detailed explanation, readers are referred to author’ review paper on MEMS microphone [35]. The noise floor affects the minimum detectable level of induced pressure from the sound waves. The most recent technique to reduce it is by employing double diaphragms or double backplates to create a differential capacitive sensing scheme [27,46,53]. Other than that, several groups manage to reduce the noise floor with a single diaphragm and backplate by controlling the air damping. They manage to achieve the noise floor of 39 dB [64], 30.5 dB [83], 35 dB [88] and 33.5 dB [56].

## 6. Future Research Direction for MEMS Capacitive Microphone

What direction of research that should be pursued next? In order to answer this million-dollars question, let us take a closer look at Table 1, especially the articles that have been published in the last five years. Based on those works, we list the possibilities herein. It should be noted that the predictions are limited to MEMS-based research activities. There are other field of research that are closely related to the development of microphones, for example, signal processing and integrated circuit design. Those are not covered here.

The first direction is the employment of new materials to make the acoustic diaphragm. We have seen recent works that use graphene [115,119,125,126,128], silicon carbide (SiC) [120], and composite materials [116,127]. Graphene is employed as researchers are ‘riding on the wave’ of this material. while SiC and composite materials are chosen due to their superior mechanical properties over Si. While the employment of new materials is the easiest route for novelty in academic publishing, the high cost associated with the exotic fabrication processes discourage industries from following through. Our recommendation is for those researchers to find a niche application for their prototypes. For example, SiC could operate at higher temperature than Si. Hence, its application as an acoustic diaphragm could be targeted for an extreme environment.

The second direction is in term of design optimization. There are many recent examples. In one, Jantawong et al. [124] introduced a stepped cavity to increase the value of the output capacitance. In another, Ganji group are pursuing the hinge or spring design to reduce the residual stress of the diaphragm [121,123]. Gharaei et al. [26] proposed a fungous coupled diaphragm to decrease the dependence of sensitivity to the effective area. In addition, two groups reported the structure that did not need a dedicated backplate [113,118]. With the wider availability of design tools in universities, we expect this direction to flourish. It should be noted, however, that design optimization is considered to be of low impact, hence published works rarely appearing in top journals. There is a way to overcome this obstacle. If those researchers combine the design optimization with better and accurate modeling of the device [133,134], the impact of their works will be bigger.

For the third direction, we have seen attempts to integrate the mechanical and electrical modules together. At stated in Section 1, Kuhnel et al. [33] and Graf et al. [34] pioneered this in early 1990s. However, their works were not followed through due to the high noise floor. Recently, we have seen publications that attempted to solve this problem [114,135,136], with some groups promoting CMOS-MEMS process [134,136] as the best solution to accomplish this goal. If these solutions are practical and proven to reduce the level of noise floor, this direction of research will be a gold mine for industry, as it pushes the cost and footprint lower.

The fourth direction is on the comprehensive testing of the prototypes. In the past, academic researchers are less interested with this direction as it is considered to be of low novelty. In industry, however, it is the opposite, as they could not release the products without passing these mandatory test procedures. Recently, we have seen two groups that are pursuing this direction. Nicollini et al. [122] developed a MEMS microphone based on a poly-Si diaphragm. They conducted comprehensive acoustical, electrical, and thermal tests to demonstrate the capabilities of their prototype. In another work, Lu et al. [134] performed comprehensive thermal test on their CMOS-MEMS microphone. Their prototype was fabricated on the Taiwan Semiconductor Manufacturing Corporation (TSMC) 0.18u process, using three aluminum layers as the diaphragm, spring, and backplate. Both recent publications are an encouraging sign. We believe that future academic works should pay more attention to proper testing of their prototypes, in particular on the reliability and repeatability aspects.

Finally, one would wonder if academia and industry are sharing similar “wish lists” for the future directions of MEMS capacitive microphone’s research activities. Therefore, we refer to Wang et al. [136], in which they present an industry view on this subject during TRANSDUCERS 2015. It is not surprising that most of the points are similar to the ones that are covered in this review. Furthermore, they emphasized a few additional items. First, the fabrication strategies to deposit non-sticking and low residual stress diaphragm. Second, the development of on-die microphones arrays to increase the signal-to-noise ratio. Third, the design of the package with shutter to protect the microphone from shock, high pressure, ESD etc. As industry prefers to patent their inventions, academic researchers should take the opportunity to publish their works on these issues.

## 7. Conclusions

MEMS capacitive microphone has been developed since 1980s. After 30 years, it still garners considerable interests in academia. The continuing attention for this device is fueled by its commercial successes. The best success story is the integration of MEMS capacitive microphone inside smart phones, as well as other IoT devices for audio and other sensing applications. We recommend academic researchers to align their future works with industry’s requirement to further develop this versatile device.

## Figures and Tables

**Table 1:** Summary of the published works on MEMS capacitive microphone. CMOS = complementary metal-oxide-semiconductor.

			Diaphragm					Backplate			Performances				
Authors [Ref] Year	Niche Application	Fabrication Method(s)	Material	Geometry and Topology	Size	Thickness (µm)	Air Gap (µm)	Electrode Material	Backplate Materials	Air Damping Mechanism	Bias Voltage (V)	Stray Capacitance (pF)	Input SPL * (dB)	Open Circuit Sensitivity (mV/Pa)	Resonant Frequency (kHz)
Hohm et al. [36] (1989)	none	bulk, back etching, bonding	Si_3_N_4_	Square flat	0.8 × 0.8 mm^2^	0.15	2.0	Al	SiO_2_	none	28	6.0	88	9	N/A
Bergqvist et al. [37] (1990)	none	bulk, back etching, bonding	Si	Square flat	2.0 × 2.0 mm^2^	5.0	4.0	Al	Glass/ Si	103 acoustic holes on back chamber	16	3.5	94	13	24
Kuhnel et al. [38] (1992)	none	bulk, back etching, bonding	Si_3_N_4_	Square flat	0.8 × 0.8 mm^2^	0.15	2.0	Al	SiO_2_/ Si	perforated backplate	28	3.0	94	1.8	30
Scheeper et al. [39] (1992)	hearing aid	surface, Plasma-enhanced chemical vapor deposition (PECVD)	Si_3_N_4_	Square flat	0.6 × 0.6 mm^2^	1.0	2.2	Ti/Au	SiO_2_	acoustic holes (120–525 holes/mm^2^)	16	7	N/A	2	14
Bourouina et al. [40] (1992)	none	bulk, anodic bonding	p+ silicon.	Square flat	1.0 × 1.0 mm^2^	1.0	N/A	Al	Si/Al	500 acoustic holes on backplate	20	N/A	N/A	3.5	120
Bergqvist et al. [41] (1994)	hearing aid	bonding, back-etching, surface micromachine	SiO_2_/Si	Square flat	2.0 × 2.0 mm^2^	5.0	2.9	Al	SiO_2_/ Si	400 holes/mm^2^ on backplate, hole diameter is 30 µm	10	4.3	94	15	17
Bergqvist et al. [42] (1994)	none	surface micromachine	Si	Square flat	1.8 × 1.8 mm^2^	4.0	3.0	Copper	Ti-Au/Copper	400 holes/mm^2.^, where holes diameter is 30 µm	28	2.9	43	1.4	47
Scheeper et al. [43] (1994)	hearing aid	surface micromachining, no bonding	Si_3_N_4_	Square flat	2.0 × 2.0 mm^2^	1.0	1,2,3	N/A	Si_3_N_4_	120–525 acoustic holes per mm^2^	5	6.6	30	10	14
Schellin et al. [44] (1994)	none	bulk micromachining	Si	Square flat	1.0 × 1.0 mm^2^	1.0	N/A	Al	Si	N/A	N/A	N/A	N/A	N/A	N/A
Donk et al. [45] (1994)	none	N/A	Si_3_N_4_	Square flat	6.0 × 6.0 mm^2^	2.0	40	N/A	Si_3_N_4_	N/A	N/A	5	N/A	N/A	N/A
Bay et al. [46] (1996)	hearing aid	bulk, back etching, anodic bonding	Si_3_N_4_	Square flat	2.0 × 2.0 mm^2^	0.2	0.4	N/A	Si	pillars at the center area of backplate electrode	N/A	N/A	N/A	N/A	N/A
Ning et al. [47] (1996)	none	bulk, surface, plasma dry etching	Si_3_N_4_	Square flat	20 × 20 µm^2^	0.5 - 1.0	3.1	Al	Si_3_N_4_	square perforated holes on backplate	6	9.5	N/A	7	10
Zou et al. [48] (1997)	none	bulk, back etching	Si_3_N_4_	Square corrugated	1.0 × 1.0 mm^2^	1.2	2.6	Al	Si_3_N_4_	hole volume is 3 mm^3^	10	N/A	N/A	14.2	16
Thielemann et al. [49] (1997)	none	bulk, back etching	SiO_2_/Si_3_N_4_	Square flat	1.2 × 1.2 mm^2^	0.4	3.0	Al/Au	Si	324 perforated holes on backplate	40	N/A	N/A	N/A	N/A
Hsu et al. [50] (1998)	none	N/A	Si	Square flat	2.0 × 2.0 mm^2^	N/A	N/A	N/A	Si	60 × 60 µm ^2^ acoustic holes on backplate	13	16.2	N/A	20	25
Pedersen et al. [51] (1998)	none	CMOS, surface (dry etching)	Polyimide	Square flat	2.2 × 2.2 mm^2^	1.1	3.6	Cr/Au/Cr	Polyimide	30 × 30 µm^2^ acoustic holes on backplate	2	N/A	120	10	15
Pedersen et al. [52] (1998)	none	CMOS, surface (dry etching)	Polyimide	Square flat	2.2 × 2.2 mm^2^	1.1	3.6	Cr/Au/Cr	Polyimide	30 × 30 µm ^2^ acoustic holes on backplate	4	10.1	120	10	15
Bay et al. [53] (1999)	hearing aid	surface, bulk micromachining	Si3N4	Square flat	2.2 × 2.2 mm^2^	0.2	0.4	N/A	Si	perforated backplate	N/A	N/A	N/A	27	N/A
Kabir et al. [54] (1999)	none	bulk and surface micromachining, electroplating technique	p+ silicon	Square flat	850 × 850 µm^2^	3.0	2.2	Au	Au	perforated backplate	9	2.4	N/A	9.77	N/A
Buhrdorf et al. [55] (2000)	ultrasonic	bulk, electroplating	poly-Si	Square flat	0.8 × 0.8 mm^2^	1.0	2.0	N/A	Nickel	perforated backplate	8	N/A	N/A	N/A	110
Torkkeli et al. [56] (2000)	none	bulk, surface micromachining	poly-Si	Square flat	1.0 × 1.0 mm^2^	0.8	1.3	Al	Si	acoustic hole size = 2 × 2 µm ^2^, perforated hole pitch = 10 µm	3	11	N/A	4	12
Brauer et al. [57] (2001)	none	bulk, surface micromachining, bonding	poly-Si	Circular flat	d = 800 − 1200 µm	0.4	N/A	N/A	Si	N/A	4.5	N/A	120	3.2	100 Hz
Hansen et al. [58] (2000)	ultrasound in air and water	N/A	Si_3_N_4_	Rectangular flat	0.1 × 0.8 mm^2^	1.3	1.0	N/A	N/A	N/A	N/A	N/A	N/A	N/A	300
Li et al. [59] (2001)	none	bulk, surface micromachining, bonding	Si	Square corrugated	1.0 × 1.0 mm^2^	1.2	2.6	Al	Si	40 × 40 holes on backplate, the dimension of each hole is 10 × 10 µm	5	1.64	N/A	10	20
Mullenborn et al. [60] (2001)	hearing aid	N/A	Si	Square flat	2.0 × 2.0 mm^2^	0.4	1.0	N/A	Si	N/A	1.5	N/A	24	5	N/A
Noble et al. [61] (2001)	ultrasound	surface micromachining	Si_3_N_4_	Square flat	5.0 × 5.0 mm^2^	0.5	2.0	AlSi	AlSi	N/A	20	N/A	N/A	1.4	N/A
Kronast et al. [62] (2001)	none	bulk, surface micromachining, bonding	Si_3_N_4_	Square flat	2.0 × 2.0 mm^2^	0.3	1.3	Al	Si	acoustic hole density = 123 holes/mm2, holes size = 60 × 60 µm^2^	6	N/A	37.7	11	25
Rombach et al. [63] (2002)	hearing aid	bulk, surface micromachining	SiN & B++ poly Si	Square flat	2 × 2 mm^2^, 1 × 1 mm^2^	0.5	0.9	Cr/Pt	Si	perforated double backplate	1.5	N/A	100	13	20Hz
Kressmann et al. [64] (2002)	none	bulk, back etching, bonding	SiO_2_/Si_3_N_4_	Square corrugated	1.0 × 1.0 mm^2^	0.6	2.0	Al	Si	144 holes, each has area of 35 × 35 µm^2^, 80 µm distance between each hole	N/A	25	39	2.9	10
Sim et al. [65] (2002)	none	patterning	parylene-C	Circular corrugated	d = 4.3 mm	3.0	N/A	N/A	N/A	N/A	N/A	N/A	N/A	NA	N/A
Jing et al. [66] (2002)	none	N/A	Si / Si3N4	Circular corrugated	N/A	N/A	N/A	Al	Si	N/A	14	N/A	N/A	40	15
Miao et al. [67] (2002)	none	bulk micromachining	poly-Si	Square flat	1.0 × 1.0 mm^2^	3.0	N/A	Al	slotted Al/nitride	acoustic holes on backplate	N/A	N/A	N/A	10	15
Chen et al. [68] (2003)	none	bulk micromachining, deep reactive ion etching (DRIE)	Si / Si3N4	Circular corrugated	d = 1.0 mm	0.6	2.5	Al	Si	N/A	14	N/A	N/A	40	N/A
Scheeper et al. [69] (2003)	none	bulk micromachining, bonding	SiN	Square flat	2.0 × 2.0 mm^2^	0.5	20	Cr/Au	Si	4 acoustic holes on backplate	N/A	N/A	N/A	22	N/A
Tajima et al. [70] (2003)	none	bulk, bonding	single crystalline Si	Square flat	2.0 × 2.0 mm^2^	5.0	15	N/A	Si	acoustic holes on backplate	48	N/A	N/A	4.4	24
Wang et al. [71] (2003)	none	bulk, surface micromachining	poly-Si	Square corrugated	1.0 × 1.0 mm^2^	1.3	2.6	Al	Si	80 × 80 µm ^2^ acoustic holes on backplate	6	N/A	N/A	20.8	N/A
Hansen et al. [72] (2004)	wide-band operation	bulk, surface micromachining	Si_3_N_4_	Rectangular flat	70 × 190 µm ^2^	0.4	N/A	Al	Si	N/A	5.8	N/A	63.6	N/A	100
Ning et al. [73] (2004)	none	bulk, surface micromachining	Si_3_N_4_	Square flat	1.5 × 1.5 mm^2^	0.5	1.5	Al	Si	40 × 40 µm ^2^ acoustic holes	8.3	N/A	N/A	5.6	20
Wang et al. [74] (2004)	none	bulk, surface micromachining	poly-Si	Square corrugated	1.0 × 1.0 mm^2^	1.3	2.6	Al	Si	N/A	6	N/A	N/A	9.8	N/A
Wang et al. [75] (2004)	none	bulk, surface micromachining	poly-Si	Square corrugated	1.0 × 1.0 mm^2^	1.2	2.6	Al	Si	N/A	5	N/A	50	16.4	20
Sezen et al. [76] (2005)	bio-medical	N/A	Si_3_N_4_	Circular flat	d = 400 µm	1.5	0.8	Al	Si	N/A	N/A	N/A	N/A	N/A	N/A
Ko et al. [77] (2006)	none	bulk, surface micromachining	doped-polySi	Square flat	1.5 × 1.5 mm^2^	0.7	2.4	Au/Ti and Pt/Ti	Si	acoustic holes on backplate with stopper	5	N/A	N/A	5.17	15
Kim et al. [78] (2006)	none	N/A	Al	Square hinge	1.5 × 1.5 mm^2^	N/A	N/A	Al	SiN/Al/SOI	N/A	25	N/A	N/A	N/A	20
Weigold et al. [79] (2006)	none	bulk	Si	Circular flat	N/A	N/A	3.0	N/A	Si	N/A	N/A	N/A	N/A	4.4	N/A
Dehe et al. [80] (2007)	hand free & hearing aid	bulk, surface micromachining	Si	Circular corrugation edge	d = 1 mm	0.4	2.0	N/A	Si	perforated backplate	2	N/A	N/A	11.2	4
Kim et al. [81] (2007)	portable terminals	N/A	Au	Square hinge	1.5 × 1.5 mm^2^	0.6	1.0	N/A	Si	N/A	1	N/A	N/A	0.01	13
Goto et al. [82] (2007)	none	bulk micromachining, bonding	Si	Square flat	2.0 × 2.0 mm^2^	8.0	varied up to 100 µm	Al	Si	N/A	48	N/A	122	6.6	20
Iguchi et al. [83] (2007)	none	bulk micromachining, bonding	Si	Square flat	2.1 × 2.1 mm^2^	8.0	10	Al	Si	10 × 10 µm^2^ acoustic holes on backplate	39	N/A	134	2.5	20
Kwon et al. [84] (2007)	none	bulk micromachining and Si DRIE	Si_3_N_4_	Square flat	2.5 × 2.5 mm^2^	0.5	9.0	Au/Ni/Cr	Si	50–60 µm radius circular acoustic holes on backplate	28	N/A	120	0.0089	5
Martin et al. [27] (2007)	aeroacoustic measurement	bulk, surface micromachining	Si	Circular flat	d = 0.46 mm	2.25	2.0	N/A	Si	hole radius: 5 µm	9.3	N/A	164	0.39	20
Kasai et al. [85] (2007)	none	4 corner supported diaphragm	poly Si	Square flat	1.2 × 1.2 mm^2^	N/A	N/A	N/A	Si	N/A	12	N/A	N/A	8.8	10
Chen et al. [86] (2008)	none	modeling and simulation	Si	Circular flat	d =560 µm	N/A	4.0	N/A	N/A	acoustic holes with diameter of 4 µm	11	N/A	100	17.7	20
Ganji et al. [87] (2008)	none	surface micromachining	Al	Square perforated	0.5 × 0.5 mm^2^	3.0	1.0	n+ backplate electrode	Si	20 × 20 µm^2^ acoustic holes	105	N/A	N/A	0.2	20
Her et al. [88] (2008)	none	bulk, surface micromachining	Si	Circular flat	d = 670 µm	1.0	3.0	Cr/Au	Si	perforated backplate	6	N/A	94	7.9	10
Hall et al. [89] (2008)	none	N/A	Si	Circular flat	d = 1.5 mm	2.3	3.0	N/A	Si	perforated backplate	N/A	N/A	N/A	N/A	20
Kaur et al. [90] (2009)	none	SOI	Si	Square flat	0.5 × 0.5 mm^2^	10 - 20 nm	0.1 to 1.95	N/A	N/A	N/A	0.04	N/A	N/A	N/A	20
Ganji et al. [91] (2009)	none	surface micromachining	Al	Square perforated	0.5 × 0.5 mm^2^	3	1.0	n+ backplate electrode	Si	holes size of 20 × 20 µm ^2^, distance between holes is 80 µm	105	2.12	N/A	0.2	20
Ganji et al. [92] (2009)	none	simulation	Al	Square slotted	2.43 × 2.43 mm^2^	3.0	1.0	n+ backplate electrode	Si	N/A	105	N/A	N/A	N/A	528
Lee et al. [93] (2009)	none	surface micromachining	Au	Circular flat	d = 300 µm	N/A	2.0	Ti/Al/TiN	Si	N/A	5	1.87	N/A	0.57	N/A
Leinenbach et al. [94] (2010)	none	bulk	Si	Circular flat	d = 0.6 mm	N/A	N/A	N/A	Si	perforated backplate	N/A	N/A	N/A	N/A	12
Ganji et al. [95] (2010)	none	surface micromachining	Al	Square slotted	N/A	3.0	1.3	Al	Si	perforated diaphragm	N/A	17.5	N/A	N/A	N/A
Yang et al. [96] (2010)	none	N/A	Si_3_N_4_	Circular flat	d = 450 µm	1.5	2.75	Al/ Au	Si_3_N_4_	N/A	12	N/A	N/A	14	N/A
Mohamad et al. [97] (2010)	none	Poly Multi-Users MEMS Process (MUMPS)	Poly-Si	Square flat spring	4.0 × 4.0 mm^2^	4	4	Au	Poly-Si	50 holes on backplate	3	N/A	N/A	4.67	10.2
Chan et al. [98] (2011)	none	surface micromachining	poly Si	Circular spring	d = 1 mm	3.0	2.0	N/A	Si	perforated rigid backplate	N/A	1.81	94	12.63	24.9
Chiang et al. [99] (2011)	mobile phones, laptops, hearing aids	N/A	Si	Rectangular flat	1444 × 1383 µm ^2^	N/A	N/A	N/A	N/A	N/A	N/A	N/A	94	N/A	20
Huang et al. [100] (2011)	none	bulk, surface micromachining	Corrugated Al	Circular corrugated	d = 800 µm	1.1	4.2	N/A	Si	air holes diameter: 20 µm	6	0.7	N/A	7.9	10
Jawed et al. [101] (2011)	none	bulk, surface micromachining	Si	Square flat	N/A	N/A	N/A	N/A	Au	N/A	N/A	1.64	55	10	9
Je et al. [102] (2011)	none	surface micromachining	Al	Circular flat center-hole	d = 500 µm	1.0	2.0	Ti/Al	SiO_2_/Al/SiN	5 holes at the center of diaphragm, where diameter of each hole is 12 µm	6	N/A	N/A	N/A	20
Kasai et al. [103] (2011)	none	bulk, surface micromachining	Si	Square flat dual channel	N/A	N/A	N/A	N/A	Si_3_N_4_ / Si	N/A	N/A	N/A	N/A	5.6	20
Lee et al. [104] (2012)	none	bulk, surface micromachining	Si_3_N_4_	Circular flat	d = 600 µm	0.9	2.45	Al	SiO_2_ / Si_3_N_4_	1668 acoustic holes on backplate, where hole radius = 4 µm.	10.4	1.02	N/A	3.75	18
Ahmadnejad et al. [105] (2013)	none	simulation	Al	Square perforated	2.43 × 2.43 mm^2^	1.0	1.0	n+ backplate electrode	Si	16 holes on diaphragm, side length = 20 µm	2.3	N/A	N/A	6.916	N/A
Chao et al. [106] (2013)	mobile device	bulk, surface	poly Si	Square flat	2.0 × 2.0 mm^2^	1.1	3.75	N/A	Si	perforated backplate	4.5	2.4	N/A	1.7	10
Je et al. [107] (2013)	none	surface micromachining, CMOS	Al/Si_3_N_4_/Al	Circular flat	d = 500 µm	1.0	2.5	Al	Al/Si_3_N_4_/Al	perforated backplate	6	N/A	N/A	10.37	20
Kuntzman et al. [108] (2014)	none	surface micromachining	poly Si	Circular flat	d = 630 µm	2.25	11	N/A	Si	air volume in the cavity with radius of 315–504 µm	100	0.25	N/A	0.167	230
Lee et al. [109] (2014)	none	simulation	Si	Square flat	900 × 900 µm ^2^	1.0	3	Ti/Au	Si	Acoustic holes with diameter of 24 µm.	12	N/A	N/A	9	79.4
Lee et al. [110] (2014)	none	bulk, eutectic bonding	Si_3_N_4_	Circular flat	d = 2 mm	1.0	3.0	Ti/Au	Si	acoustic holes cover 18% of backplate	12	N/A	N/A	13	10
Grixti et al. [111] (2015)	none	N/A	Si	Square flat	675 × 675 µm^2^	0.5	2.0	Au	Si	holes-to-backplate ratio = 0.33	6	1.53	139	8.4	1
Kuntzman et al. [112] (2015)	ultrasonic	N/A	Si	Circular flat	d = 630 µm	2.3	0.3	N/A	Si	square holes on backplate	50	N/A	N/A	10	18.8
Lo et al. [113] (2015)	none	bulk, surface micromachining	Si	Circular flat	d = 600 µm	1.6	1.6	N/A	No backplate	N/A	N/A	N/A	N/A	N/A	1
Shin et al. [114] (2015)	none	electret substrate: bulk, surface	Si	Circular flat	d = 1.2 mm	5.0	5.0	Cr/Au	Si	110 µm diameter acoustic holes	N/A	N/A	107	N/A	20
Todorovic et al. [115] (2015)	none	bulk, surface micromachining	Multilayer graphene	Circular flat	d = 12 mm	0.025	18.6	N/A	N/A	N/A	200	N/A	90	50	6.5
Gharaei et al. [26] (2016)	aerospace application	simulation	Si	Circular flat	d = 660 µm	230	2.0	N/A	Si	367 acoustic holes on backplate	11	1.15	N/A	0.478	100
Lee et al. [116] (2016)	none	bulk, surface micromachining	TiN/Si_3_N_4_/TiN	Circular flat	d= 0.65 mm	0.6	1.6	Al	Si_3_N_4_	acoustic holes on backplate, radius: 4 µm	11.1	0.23	N/A	5.3	10
Manz et al. [117] (2017)	none	N/A	Si	Rectangular flat	500 × 800 µm ^2^	N/A	0.5	N/A	N/A	N/A	N/A	N/A	73	12.5	35
Mao et al. [118] (2017)	none	CMOS	Pure dielectric-film	Circular flat	d = 300 µm	N/A	N/A	N/A	No backplate	N/A	13.5	N/A	N/A	0.6	22
Woo et al. [119] (2017)	hearing aid	Bulk micromachining	Graphene / Polymethylmethacrylate (Acrylic) or PMMA	Circular flat	d = 4.0 mm	N/A	10	Au	Ti	N/A	N/A	N/A	90	100	7.0
Zawawi et al. [120] (2017)	detect poisonous gas	Finite element analysis (FEA) simulation	3C-SiC	Square flat	1.0 × 1.0 mm^2^	1.0	3.0	N/A	N/A	perforated backplate	N/A	N/A	N/A	N/A	36
Sedaghat et al. [121] (2018)	none	FEA (simulation)	Al	Square perforated	0.5 × 0.5 mm^2^	3.0	1.0	N/A	Si	perforated diaphragm area is 0.0144 mm^2^	1.35	N/A	N/A	6.677	21.504
Nicollini et al. [122] (2018)	none	CMOS	Poly-Si	Rectangular flat	0.5 × 1.0 mm	N/A	5.6	N/A	N/A	acoustic holes on the backplate	N/A	N/A	120	12.58	20
Ganji et al. [123] (2018)	none	bulk micromachining using SOI wafer	Si	Square perforated	0.3 × 0.3 mm^2^	5.0	1.0	N/A	Si	5 × 5 µm holes size on perforated diaphragm	5	N/A	N/A	2.46	60
Jantawong et al. [124] (2019)	none	bulk micromachining	Poly-Si	Circular flat	d = 930 µm	0.8	3.5	Al	Si	perforated backplate	N/A	N/A	123	N/A	N/A
Wittmann et al. [125] (2019)	none	CMOS	Graphene	Circular flat	d = 40 µm	N/A	N/A	Au	Si	N/A	1.5	N/A	N/A	1.051	100
Mustapha et al. [126] (2019)	none	bulk micromachining	Graphene	Circular flat	d = 40 µm	0.5	0.2	Cr/Au	Si	N/A	3.0	N/A	N/A	0.035	20
Auliya et al. [127] (2019)	none	FEA simulation	Si/SiC/tungsten	Circular corrugated	d = 2.0 mm	18	18	N/A	N/A	N/A	41	N/A	N/A	0.15	70
Malik et al. [3] (2019)	hearing aid	N/A	Si_3_N_4_	Circular flat	area = 7850 µm ^2^	2.0	N/A	N/A	Si	perforated backplate	4.0	N/A	N/A	0.086	10
Wood et al. [128] (2019)	none	bulk micromachining	Graphene/PMMA	Circular flat	d = 3.5 mm	0.2	8.0	Al	SiO_2_/Si	N/A	1.0	N/A	80	10	20

* Input SPL (dB) refers to maximum sound pressure level. Some papers put 94 dB. This is the standard value that is used to test the sensitivity of their prototypes. The detailed explanation can be found in Section 5.3 of this paper. Note: N/A refers to data about specific parameter that is not provided by the authors. For example, many articles only present the diaphragm, so information on backplate are labeled as N/A. Similarly, not all papers provide complete information on the performance parameters.
